# Structural and dynamic characterization of a novel in-frame indel variant in glucokinase associated with maturity-onset diabetes of the young type 2

**DOI:** 10.1210/jendso/bvag250

**Published:** 2026-10-23

**Authors:** Hafiz Muhammad Jafar Hussain, Maria Daniela Hurtado Andrade, Laura Duncan, Young-In Chi, Raul Urrutia, Filippo Pinto e Vairo

**Affiliations:** Center for Individualized Medicine, Mayo Clinic, Rochester, MN 55905, USA; Department of Quantitative Health Sciences, Mayo Clinic, Rochester, MN 55905, USA; Division of Endocrinology, Diabetes, Metabolism, and Nutrition, Department of Medicine, Mayo Clinic, Jacksonville, FL 32224, USA; Research and Translational Genetic Counseling Unit, Clinical Research, Mayo Clinic, Jacksonville, FL 32224, USA; Mellowes Center for Genomic Sciences and Precision Medicine, Medical College of Wisconsin, Milwaukee, WI 53226, USA; Department of Molecular Pharmacology and Experimental Therapeutics, Mayo Clinic Comprehensive Cancer Center, Mayo Clinic, Phoenix, AZ 85054, USA; Department of Medicine, Division of Gastroenterology and Hepatology, Mayo Clinic, Rochester, MN 55905, USA

**Keywords:** MODY, glucokinase, molecular dynamics, variant interpretation

## Abstract

Human glucokinase (GCK) catalyzes the rate-limiting step of β-cell glycolysis and serves as a critical glucose sensor in the regulation of glucose homeostasis. Variants in *GCK* are a leading cause of maturity-onset diabetes of the young (MODY), accounting for 30% to 50% of MODY cases depending on the population studied. Clinical management of GCK-MODY during pregnancy poses particular challenges, as the fetal genotype, which influences fetal insulin secretion and growth, is typically unknown in utero. In this report, we present a case of GCK-MODY identified during pregnancy and provide a mechanistic assessment of the functional impact of a variant of uncertain significance (VUS). We identified a novel heterozygous in-frame indel VUS (NM_000162.5:c.311_325delinsTCCCCGAGGACG: p.(Lys104_Ser109delinsIleProGluAspAla)) in *GCK* in a patient initially managed as type 2 diabetes. The affected region lies within a β-strand of the small domain that undergoes a major conformational transition during enzyme activation and is known to be intolerant of sequence variation. Structural modeling and molecular dynamics simulations reveal that this variant disrupts normal movement of the small domain, alters coordinated residue fluctuations, and impairs inter-domain communication during the activation process. These perturbations are accompanied by pronounced protein destabilization and a trend toward weaker adenosine triphosphate (ATP) interaction, consistent with a loss-of-function mechanism. These findings provide a mechanistic framework for interpreting this novel variant, expanding the known genotypic spectrum of GCK-MODY, and may inform future structure-guided strategies for GCK-targeted therapeutics.

Glucokinase (GCK), also referred to as hexokinase IV, is a member of the hexokinase enzyme family and plays a central role in glucose homeostasis. It catalyzes the initial, rate-limiting step of glycolysis in the liver and pancreatic β-cells, mediating the adenosine triphosphate (ATP)-dependent conversion of glucose to glucose-6-phosphate (G6P) (1, 2). This reaction is critical for initiating insulin secretion in pancreatic β-cells and promoting glycogen synthesis in hepatocytes. The central role of GCK in glucose-regulated insulin secretion underscores its importance in glucose metabolism (3).

The mechanistic basis of GCK activation and catalytic activity has been well characterized (4-6). Activation involves a substantial conformational change in the small domain as it recognizes and engages incoming glucose substrate and the ATP cofactor. The active-state conformation can be further stabilized by GCK activators that bind to the allosteric site located between the small and large domains (6, 7). In the liver, this activation process is inhibited by the hepatocyte-specific GCK regulatory protein, which binds to the allosteric site and functions as a competitive inhibitor of glucose binding (8, 9). Molecular dynamics (MD) simulation studies support this cooperative conformational shift during activation, revealing a unidirectional circulation between the inactive and active states and a role for glucose in modulating its own binding pose (6, 10, 11). In addition to its primary expression in the liver and pancreas, GCK is present in several other tissues, including the intestine, hypothalamus, pituitary gland, lung, and spleen (2). Unlike other hexokinase isoforms, GCK exhibits a relatively low affinity for glucose and is not subject to feedback inhibition by its product, G6P (3). These unique kinetic properties enable GCK to function as a physiological glucose sensor, a role that is essential for maintaining glucose balance in vertebrates. Disruptions in GCK function can impair glycolysis, a critical metabolic pathway, and lead to diabetes mellitus. Therefore, GCK has emerged as a promising therapeutic target, with GCK activators offering an alternative strategy for restoring glycemic homeostasis in diabetic patients (12-15). Accurate genetic diagnosis is essential for guiding appropriate clinical management, particularly for distinguishing monogenic forms of diabetes from more common types.

Maturity-onset diabetes of the young (MODY) is a monogenic form of diabetes mellitus caused by variants in several genes, including *HNF1A*, *HNF4A*, *GCK*, *HNF1B*, *PDX1*, among others. Notably, *GCK* variants alone account for 30% to 50% of MODY cases depending on the population studied (16). Dominant and recessive variants in *GCK* have been associated with MODY type 2 (MODY2) and neonatal diabetes, respectively (17-19), and GCK-MODY is identified in approximately 2% (range 0-6%) of women with gestational diabetes (20-23). Such pregnancies are associated with an increased risk of complications including hyperglycemia, fetal macrosomia, and pre-eclampsia. Moreover, a few missense gain-of-function variants in *GCK* have been associated with hyperinsulinism and increased body weight (24-26).

Clinical management is particularly challenging because the fetal genotype, typically unknown in utero in the absence of invasive testing, directly determines fetal growth, and therefore the appropriateness of maternal blood sugar control.

Here, we describe a case referred to the Division of Endocrinology at Mayo Clinic for evaluation of mild but persistent hyperglycemia in a woman with a normal-weight body mass index (BMI). She was initially diagnosed with type 2 diabetes (T2D); however, an atypical clinical phenotype including a poor response to metformin therapy and weight loss, together with a negative biochemical evaluation for autoimmune diabetes raised the possibility of MODY. This suspicion was further reinforced by a family history notable for early-onset diabetes in 2 first-degree relatives. In the context of a newly discovered second pregnancy shortly after initial evaluation, the patient was referred for genetic evaluation.

Genetic testing identified a novel in-frame indel variant of uncertain significance (VUS) in *GCK*. To assess the functional significance of this variant, we performed structural modeling and MD simulations of the mutant protein alongside benign control variants, providing mechanistic evidence for the pathogenic impact of this previously unreported variant.

## Materials and methods

### Patient data and ethical approval

Clinical history, genetic analysis data, and sociodemographic information were obtained from the patient’s medical records at Mayo Clinic, Jacksonville, Florida, United States. This study was approved by the Mayo Clinic Institutional Review Board (IRB-19-003389). Written informed consent was obtained for clinical investigation and publication of the variant findings.

### Variant classification

The identified variant was classified according to the American College of Medical Genetics and Genomics/Association for Molecular Pathology guidelines for sequence variant interpretation. The variant is absent from gnomAD v4.1.0 (27, 28) (PM2_Supporting), is an in-frame indel that alters protein length outside a repeat region (PM4), and is supported by concordant in silico prediction of a deleterious effect (PP3; MutPred-Indel score 0.88) (29). It lies within a highly conserved, variant-intolerant segment of the small domain that harbors multiple pathogenic and likely pathogenic variants and no reported benign variation; PM1 was considered but applied conservatively in the absence of a formal enrichment analysis. On this basis the variant is classified as a VUS.

### Multiple sequence alignment

Protein sequences were retrieved from UniProt (https://www.uniprot.org/) using the following accession numbers: Human (P35557), Rhesus macaque (F6PLU3), Dog (A0A8I3RXD0), Elephant (G3TLS7), Mouse (P52792), Chicken (A0A8V0Z8U3), Xenopus tropicalis (A0A803JR65), and Zebrafish (F1RC38). Multiple sequence alignment was performed using the ClustalW online tool (https://www.genome.jp/tools-bin/clustalw).

### Structure modeling of wild-type and variant human GCK

Experimental structures of GCK in multiple conformations, from the apo inactive state to the ATP- and glucose-bound active state, have been previously determined (6, 11). We used both the fully open apo conformation (PDB: 1V4T) and the fully closed active conformation (PDB: 3FGU) as starting models. Missing regions in the crystal structures, including flexible loops spanning residues 67 to 68, 94 to 96, 141 to 142, and 157 to 179, were reconstructed using the AlphaFold-predicted structure (AF-P35557-F1-model_v4) (30). The fully active model contains the substrate glucose, cofactor ATP, and a coordinated Mg^2+^ ion in the active site. The in-frame indel variant, which introduces 5 amino acid substitutions and 1 deletion across residues 104 to 109, was modeled in both conformations using the SWISS-MODEL server (31). The 3 benign control missense variants (p.(Arg46Lys), p.(Asp217Asn), and p.(Glu279Gln)) and the 3 pathogenic control variants (p.(Gln106Pro), p.(Tyr108Asp), and p.(Tyr108His)) were also modeled using the SWISS-MODEL workflow. Building the wild-type and all variant structures with a single modeling procedure was done deliberately to eliminate model-construction differences as a source of variation. All wild-type and variant structures were then subjected to identical energy minimization and MD simulation protocols as described below.

### Protein folding energy, stability calculation, and structural perturbation analysis

The impact of each variant on protein stability was assessed by comparing folding free energies (ΔG_fold) between wild-type and mutant structures using FoldX (32). Because the case variant includes a single-residue deletion, ΔΔG was computed with the FoldX BuildModel command, which models the deletion by assigning the affected position as a gap; ΔΔG was calculated across the affected positions and averaged. Positive ΔΔG values (kcal/mol) indicate destabilization relative to wild-type. Energy-minimized structures were used for all calculations, with each variant introduced into both the inactive (open) and active (closed) wild-type conformations.

Global and local structural perturbations were quantified by measuring the positional displacement of backbone atoms between wild-type and variant structures. For global perturbation, the root mean square deviation (RMSD) of all backbone atoms across the entire catalytic domain was calculated. For local perturbation, residues within a 10 Å radius of the mutation site were selected using PyMOL (Molecular Graphics System, Schrödinger, LLC), and the least-squares RMSD of backbone atoms within this region was calculated between wild-type and variant structures using Coot (33).

### MD simulations

All MD simulations were performed using the CHARMM36 all-atom force field implemented in Discovery Studio, with a 2-fs integration time step (34). Simulations employed a simplified implicit solvent model using a distance-dependent dielectric constant of 80 at pH 7.4. No additional parameterization was applied to the ATP cofactor, Mg^2+^ ion, or glucose substrate; accordingly, the simulations are intended to capture backbone and domain-level dynamics rather than precise binding free energies. Energy minimization was performed in 2 stages: 5000 steps using the steepest descent algorithm followed by 5000 steps of conjugate gradient minimization, to relax structures obtained under the constraints of the crystal environment. For each system (wild-type and each variant), ten independent replicates were generated by varying the initial velocity seed. Each replicate was heated to 300 K over 200 ps, equilibrated for 500 ps, and subjected to a 10 ns production run under the nucleoside triphosphate (NPT) ensemble (constant number of atoms, pressure, and temperature), yielding a cumulative 100 ns of simulation time per system. Structures were recorded every 10 ps, producing 1000 frames per replicate for subsequent analysis. We note that implicit solvent simulations at this timescale are best suited for capturing relative differences in domain-scale flexibility and conformational sampling rather than absolute binding thermodynamics. The use of ten independent replicates with varied initial conditions was designed to improve conformational sampling within this framework.

### Time-dependent interaction energy and distance monitoring

Time-dependent molecular interaction energies and interatomic distances were measured from the MD trajectories using protocols implemented in Discovery Studio. For each system, the protein and specific interaction groups of interest (glucose, ATP, and key catalytic residues) were selected, and nonbonded interaction energies, comprising van der Waals and electrostatic components, were calculated from the trajectories using the CHARMM36 force field and the implicit distance-dependent dielectric solvent model. Spontaneous molecular interactions are represented by negative free energy values. Potentially damaging scores are reported as differences relative to the wild-type. The positive differences in free energy values indicate weaker interactions. Interaction energies and distances were computed for each replicate and averaged across all ten replicates. Dynamic fluctuations per residue were assessed by calculating the root mean square fluctuation (RMSF), and the correlation between wild-type and variant RMSF profiles was used as a metric for evaluating disturbance in coordinated motions.

## Results

### Clinical evaluation

A 28-year-old woman with a history of T2D and polycystic ovary syndrome (PCOS). Her weight was 65.8 kg (145 lb), height was 165.1 cm, and BMI was 24.13.

At the age of 27 years, her serum C-peptide levels were low (0.67 ng/mL, normal range 0.80-3.85 ng/mL). In the setting of the low C-peptide, she underwent evaluation for type 1 diabetes antibodies, which were all negative (glutamic acid decarboxylase [GAD65], insulinoma-associated antigen 2 [IA-2], and insulin autoantibodies). She was diagnosed with T2D at age 25 in the setting of a hemoglobin A1c of 6.8%. She was initially treated with metformin, with dose escalation from 500 mg twice daily to 500 mg 3 times daily; however, her glycemic control improved only minimally despite adequate dosing and a subsequent 10-pound weight loss. Her persistently and only mildly elevated hemoglobin A1c in the setting of normal body weight was atypical for a routine presentation of T2D and prompted further evaluation at Mayo Clinic, Jacksonville, FL, United States.

To clarify the underlying etiology, beta-cell reserve and autoimmune markers were assessed. A comprehensive type 1 diabetes autoantibody panel—including GAD65, IA-2, and insulin autoantibodies—was negative, and the serum C-peptide, although marginally below the reference range, was detectable, indicating retained endogenous insulin secretion rather than the profound insulinopenia expected in type 1 diabetes. Together, these findings argued against autoimmune diabetes as the underlying mechanism and raised consideration for monogenic etiology. This hypothesis was further reinforced by a multigenerational family history, including a maternal half-sister diagnosed with diabetes at age 12 and a diagnosis of prediabetes in the proband’s own daughter, a pattern more consistent with autosomal dominant inheritance than with typical T2D. Given this clinical and family history profile and particularly in the context of a newly discovered (second) pregnancy shortly after initial evaluation the patient was referred for genetic evaluation.

Genomic testing for MODY identified a heterozygous in-frame indel VUS in *GCK*. Due to the uncertainty regarding the variant, diabetes management during pregnancy was guided by the suspicion of GCK-MODY, in which GCK functions as the pancreatic glucose sensor and resets the threshold for insulin secretion to a higher set point. Fetal genotype was recognized as the key determinant of risk since an affected fetus shares the maternal glucose set point and perceives maternal hyperglycemia as normal, resulting in appropriate growth without the need for treatment escalation, whereas an unaffected fetus is exposed to glucose levels above its own normal threshold, risking fetal hyperinsulinemia, accelerated growth, and macrosomia.

Given this genotype-dependent divergence, invasive fetal genetic testing was discussed with the patient’s obstetrician but ultimately not pursued. Serial fetal abdominal circumference measurement beginning in the second trimester was instead recommended as a noninvasive surrogate for fetal genotype and growth trajectory, with insulin recommended in the setting of disproportionate growth (abdominal circumference >75th percentile) and a planned cesarean delivery at 38 weeks if insulin was initiated (21, 35). Because metformin is generally ineffective in GCK-MODY, discontinuation was recommended, with a plan to monitor glucose off therapy; reinstitution was held in reserve only if hyperglycemia worsened beyond the mild, nonprogressive range expected in GCK-MODY, recognizing that the variant remained of uncertain significance and that a contribution from coexisting insulin resistance related to PCOS could not be excluded.

Despite reassuring fetal growth throughout pregnancy, maternal glucose remained above the target. Given the patient’s history of a prior pregnancy at age 17 complicated by pre-eclampsia, placental abruption, and an emergent classical cesarean section at 26 weeks of gestation, she experienced significant anxiety and expressed a strong preference to initiate insulin sooner; metformin was therefore reinitiated and Neutral Protamine Hagedorn insulin was added. The insulin regimen was titrated throughout the remainder of pregnancy and was well tolerated, with only infrequent, mild hypoglycemia. She underwent a scheduled cesarean delivery at 37 weeks and delivered a healthy newborn with growth parameters appropriate for gestational age. Following delivery, insulin was discontinued, metformin was continued, and she was discharged in stable condition. She subsequently had a third pregnancy at age 31, during which she had a clinical course similar to her second pregnancy. Insulin was initiated during pregnancy and discontinued upon delivery. The proband also had a history of sensorineural hearing loss. Because of the combination of diabetes, hearing loss, and a maternal-line family history, the mitochondrial genome was reviewed and the m.3243A>G variant was absent, making maternally inherited diabetes and deafness unlikely, and the hearing loss is considered unrelated to the GCK-MODY phenotype.

### Genetic findings

A custom 91-gene monogenic diabetes panel (Table S1) using short-read genome-backbone-based sequencing performed in a CLIA-certified laboratory identified a heterozygous in-frame indel VUS in *GCK* (NM_000162.5: c.311_325delinsTCCCCGAGGACG: p.(Lys104_Ser109delinsIleProGluAspAla)) (Table S1, and details are given in Supplementary Material (36)). Additional variants were not reported, and family studies could not be completed to determine the inheritance of the variant due to family preference. Analysis of the mitochondrial genome, which was included in the genome sequencing, was negative. This *GCK* variant is absent from the gnomAD v4.1.0 population database and has not been previously reported in the literature. The sequence-based predictor MutPred-Indel (31) assigned a high probability of pathogenicity (score 0.88; neutral rate < 0.5). The variant resides in a highly conserved and mutationally intolerant region of the small domain (Fig. 1A and 1B), where no benign variants have been reported. The variant spans residues 104-109 and is predicted to result in 5 amino acid substitutions and 1 deletion: Lys104→Ile, His105→Pro, Gln106→Glu, Met107→Asp, Tyr108→Ala, and deletion of Ser109. Several lines of genomic evidence support the pathogenic relevance of this region. A variant at position 106 (p.(Gln106Pro); ClinVar ID: 2576969) has been classified as likely pathogenic and associated with MODY. Multiple missense variants at position 108: p.(Tyr108Cys), p.(Tyr108Phe), and p.(Tyr108His) have been reported in association with MODY and hyperglycemia, and are classified as VUS, VUS, and pathogenic, respectively (ClinVar IDs: 2581306, 2691834, and 585918) (37-41). Furthermore, an in-frame indel in the same region (p.(Gln106_Met107delinsLeu)) has been reported in association with MODY2 (42).

**Figure 1:**
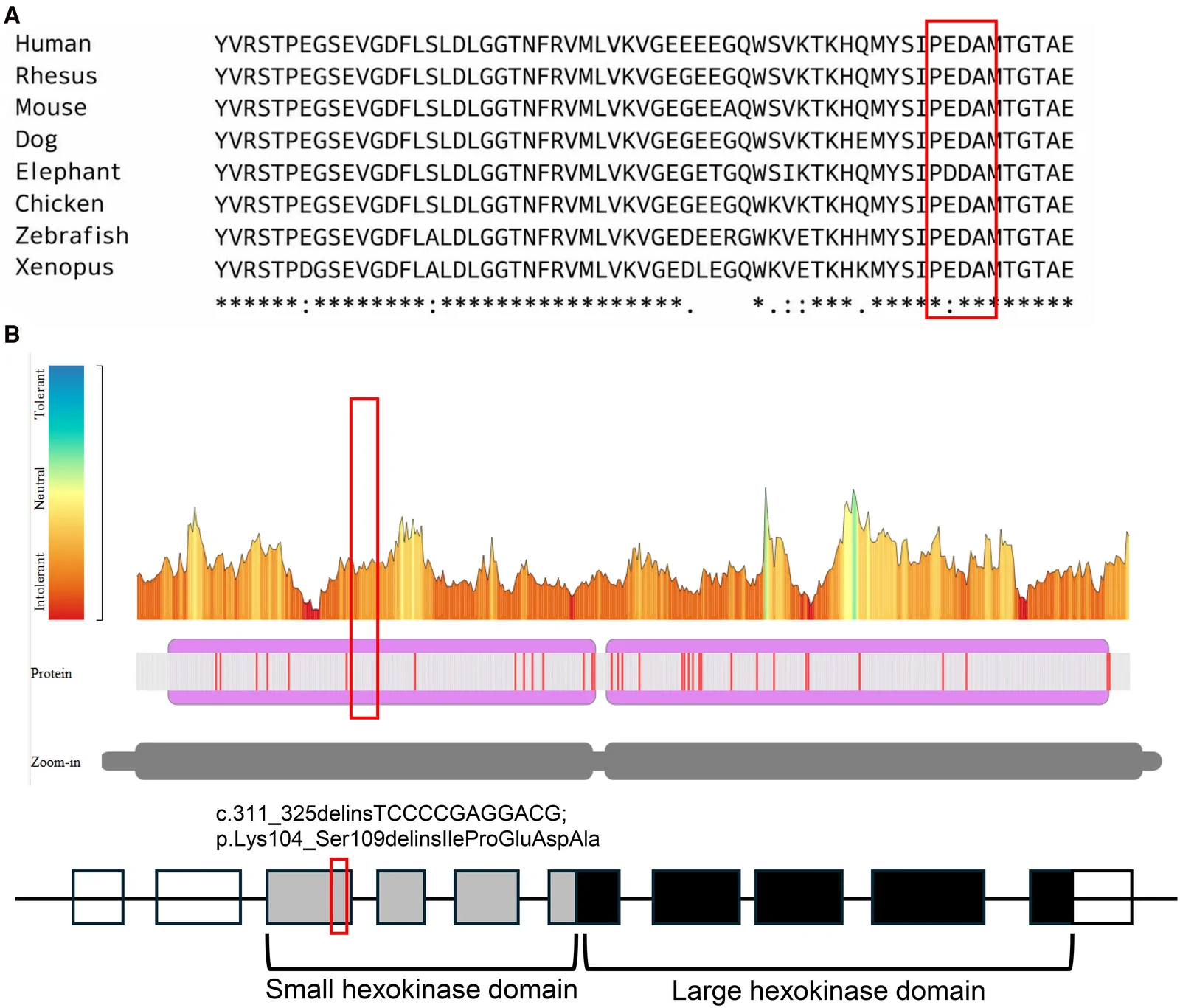
Conservation and domain context of the identified variant. (A) Multiple sequence alignment across species shows that the residues spanning the in-frame indel are highly conserved. (B) MetaDom tolerance landscape showing that the identified variant (p.(Lys104_Ser109delinsIleProGluAspAla)) falls within a mutationally intolerant region of the protein. The variant maps to the small hexokinase domain, which undergoes the major conformational transition required for GCK activation. Boxes indicate the impacted amino acids by the identified in-frame indel variant.

Collectively, these data identify residues 104 to 109 as a highly conserved, variant-intolerant region harboring multiple pathogenic and likely pathogenic variants, supporting the functional relevance of the identified variant.

### Structural and dynamic impact of the p.(Lys104_Ser109delinsIleProGluAspAla) variant

#### Protein architecture and active site

Human GCK is a monomeric allosteric enzyme composed of 2 domains: a small domain (residues 66-202 and 442-465, the latter comprising the C-terminal α13 helix) and a large domain (residues 1-65 and 203-441) (Fig. 2A and 2B). The C-terminal α13 helix serves as a pivot point for inter-domain movement, shifting outward to allow the small domain to fold inward during the activation process (6). The active site contains 2 key catalytic residues: Asp205, which acts as a general base to activate the O6 hydroxyl group of glucose, and Lys169, which functions as a general acid to protonate the γ-phosphate of ATP, with MgATP^2−^ serving as the phosphoryl donor (Fig. 2A and 2C). Time-dependent interaction energies and distances among these key elements were measured as part of the overall assessment of variation impact.

**Figure 2:**
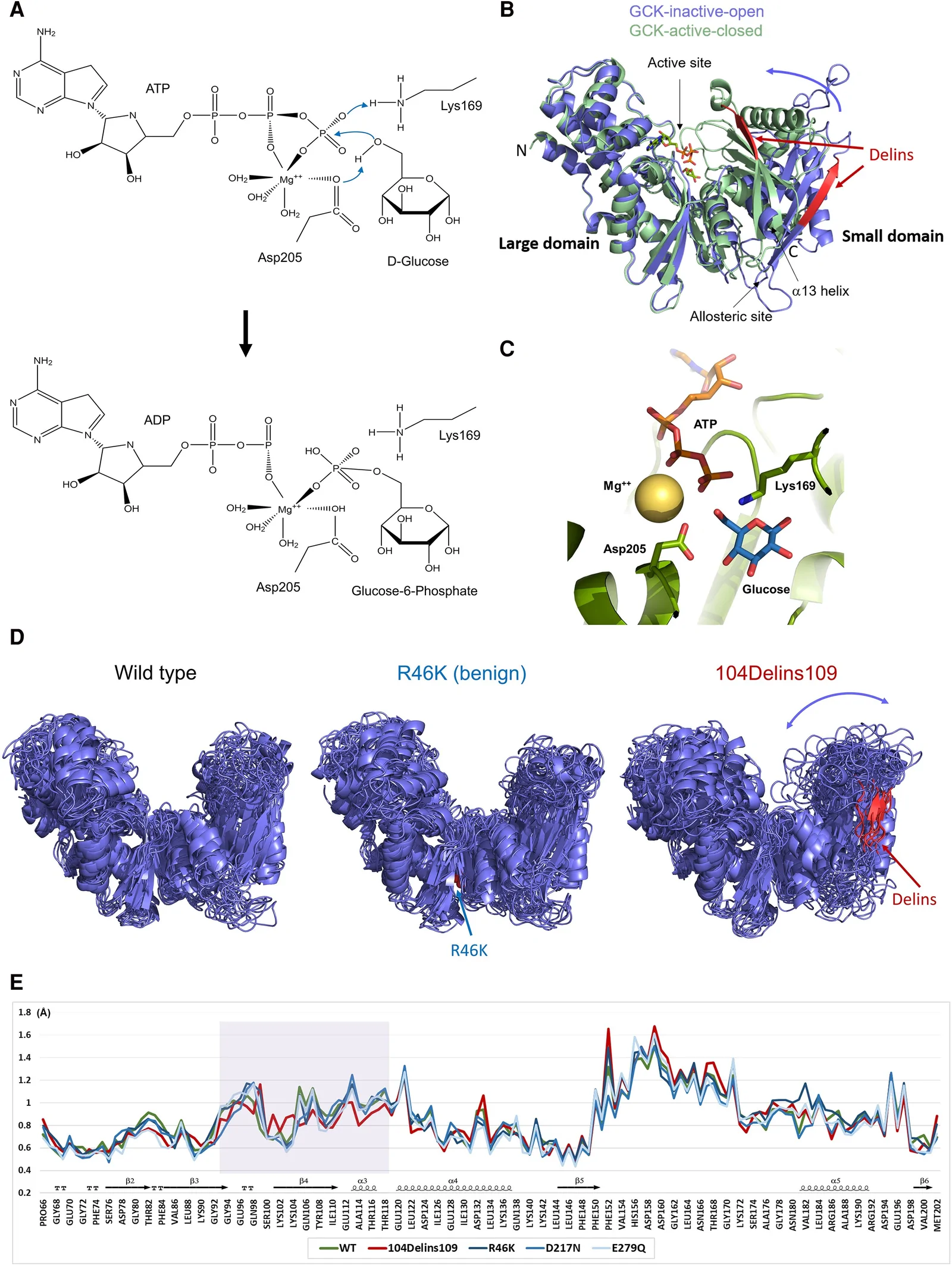
Structural and dynamic impact of the p.(Lys104_Ser109delinsIleProGluAspAla) variant on GCK. (A) Schematic representation of the GCK catalytic mechanism, illustrating the key residues involved in glucose phosphorylation. Asp205 acts as a general base to activate the O6 hydroxyl group of glucose, while Lys169 functions as a general acid to protonate the γ-phosphate of ATP. MgATP^2−^ serves as the phosphoryl donor. (B) Superposition of GCK structures in the fully open inactive (purple) and fully closed active (pale green) conformations, highlighting the large-scale conformational change of the small domain during activation. The region affected by the indel variant (residues 104-109) is shown in red in both conformations. (C) Close-up view of the GCK active site showing the spatial arrangement of the glucose substrate, ATP cofactor, Mg^2+^ ion, and key catalytic residues. (D) Superposition of the final inactive open conformations from ten independent MD simulation replicates for the wild-type protein, the representative benign control variant p.(Arg46Lys), and the p.(Lys104_Ser109delinsIleProGluAspAla) indel variant. The wild-type and benign control variant maintain consistent and structurally comparable conformations across replicates. In contrast, the indel variant produces markedly variable and distorted conformations of the small domain, including increased sampling of more closed conformations that would be expected to disrupt optimal positioning of the substrate and cofactor in the active site. (E) Superposition of root mean square fluctuation (RMSF) profiles of the inactive open conformation per residue for the wild-type and all variants. The indel variant (red) displays pronounced deviations from the wild-type fluctuation profile in the region surrounding the mutation site (shaded box), indicating disrupted local dynamics. All 3 benign control variants (p.(Arg46Lys), p.(Asp217Asn), and p.(Glu279Gln)) show fluctuation patterns closely matching the wild-type.

#### Selection of benign and pathogenic control variants

To contextualize the impact of the indel variant, 3 benign control variants were selected from ClinVar entries annotated as “benign” with relatively high allele frequencies in gnomAD: p.(Arg46Lys), p.(Asp217Asn), and p.(Glu279Gln). All 3 are surface-exposed residues located away from the active site and fully accessible to solvent and are therefore expected to have minimal structural or functional consequences. According to the experimental data deposited in the MAVE database (43), these variants exhibited GCK activities comparable to those of the wild-type protein (Table 1, column 3). In addition, we identified 3 pathogenic control variants within the affected region from the MAVE database and included them in our analyses: p.(Gln106Pro), p.(Tyr108Asp), and p.(Tyr108His). All 3 pathogenic variants exhibited reduced GCK activity. We could not identify any benign variants within the region or any tolerated in-frame indel variants.

**Table 1:** Structural and molecular dynamics-based assessment scores of the identified variants

Variant (ClinVar variant ID)	ClinVar classification	MAVE GCK activity score (baseline score 1.00)	Structure (3D)-based analysis			Molecular dynamics (4D)-based analysis						
			ΔΔGFoldX (kcal/mol)	Global perturb (Å)	Local perturb (Å)	RMSF Scorr*^b^*	RMSF Pcorr*^a^	ΔATP interaction (−645.2660 kcal/mol)	ΔGlucose interaction (−87.0010 kcal/mol)	ΔGlucoseO6- Asp205OD distance (4.4085 Å)	ΔATPPg- GlucoseO6 distance (3.4626 Å)	ΔATPPg- Lys169NZ distance (3.1315 Å)
**Inactive-open model**												
K104_S109delinsIPEDA	Case variant	NA	6.53	3.271	2.356	0.9086	0.9243	NA	NA	NA	NA	NA
R46K (1802664)	Benign	0.6988	0.19	0.086	0.287	0.9367	0.9359	NA	NA	NA	NA	NA
D217N (911631)	Benign	1.0030	0.66	0.069	0.140	0.9347	0.9359	NA	NA	NA	NA	NA
E279Q (211076)	Benign	1.2070	−0.35	0.064	0.136	0.9381	0.9396	NA	NA	NA	NA	NA
Q106P (2576969)	Pathogenic	−0.1248	5.72	0.116	0.367	0.9280	0.9295	NA	NA	NA	NA	NA
Y108D (36212)	Pathogenic	−0.3146	3.35	0.107	0.335	0.9269	0.9248	NA	NA	NA	NA	NA
Y108H (585918)	Pathogenic	−0.2427	1.89	0.071	0.192	0.9185	0.9160	NA	NA	NA	NA	NA
**Active-closed model with bound ATP, Mg^++^, and glucose**												
K104_S109delinsIPEDA	Case variant	NA	7.90	3.128	2.502	0.9047	0.9073	20.7444	1.2420	−0.3756	0.0782	0.2721
R46K (1802664)	Benign	0.6988	0.12	0.038	0.105	0.9267	0.9294	12.5973	−2.8790	0.3383	0.0363	0.2023
D217N (911631)	Benign	1.0030	0.73	0.054	0.167	0.9122	0.9181	−2.1173	−10.7364	−0.5942	0.0054	−0.0518
E279Q (211076)	Benign	1.2070	−0.32	0.021	0.071	0.9335	0.9338	−5.2203	−6.1050	−0.0411	0.1211	−0.0465
Q106P (2576969)	Pathogenic	−0.1248	5.02	0.136	0.443	0.9361	0.9342	16.0543	2.5425	0.1992	−0.0137	0.2510
Y108D (36212)	Pathogenic	−0.3146	4.58	0.073	0.223	0.9260	0.9153	12.8200	−18.9058	−0.8726	−0.1455	−0.0949
Y108H (585918)	Pathogenic	−0.2427	3.41	0.051	0.151	0.9066	0.9078	35.9816	−10.0289	−0.1874	0.0781	−0.1524

Table of individual damaging scores by each analysis. In this table, only the values using the structure (3D)-, and MD (4D)-based metrics are shown for the p.(Lys104_Ser109delinsIleProGluAspAla) in-frame indel variant and the control variants. The wild-type values are shown in parenthesis, and many damaging scores are reported as differences relative to the wild-type. The intensities of the pink shades are proportional to the degree of predicted damaging impacts. Overall, all benign control variants are predicted to be tolerated by these predictions while pathogenic control variants are predicted to be damaging. Lower RMSF correlations imply greater non-native dynamics. The positive free energies indicate weaker interactions. Structural perturbation, disturbed dynamic fluctuations, and a trend toward reduced interaction with the ATP cofactor by the indel variant are apparent.

Abbreviation: ATP, adenosine triphosphate; NA, not available.

^*a*^Pearson correlation coefficient.

^*b*^Spearman correlation coefficient.

#### Analytical framework

Impact analysis was performed using both the fully open inactive (apo) and fully closed active (ATP and glucose-bound) conformations. We applied a multilayered assessment strategy integrating metrics from the sequence level (2D), static structure level (3D), and all-atom MD level (4D). The structurally and functionally relevant parameters included protein folding energy and stability, global and local structural perturbation, and various dynamic motion-dependent metrics.

#### Protein stability and structural perturbation

The indel variant showed markedly reduced folding energy and perturbed global and local structures in both conformations (Table 1, columns 4-6). Loss of a portion of the β-strand secondary structure was observed, driven primarily by the introduction of a proline residue, whose rigid pyrrolidine ring disrupts backbone hydrogen bonding patterns that stabilize β-sheet architecture. The local structure was also slightly distorted and shortened as a consequence of the 5 amino acid substitutions and single amino acid deletion (Fig. S1 (36)). The destabilizing effect on folding energy was slightly more pronounced in the active closed conformation than in the inactive open conformation.

#### MD and domain motion

Molecular dynamics simulation data indicate that the indel variant causes aberrant dynamic motions of the small domain and disrupts local coordinated fluctuations (Fig. 2D and 2E). Superposition of the final structures from the ten replicate simulations revealed consistent and comparable conformations for the wild-type and the p.(Arg46Lys) benign variant (shown as a representative control, also see Fig. S2 for all other variants (36)). In contrast, the indel variant produced more variable and distorted conformations of the small domain, with increased sampling of slightly more closed conformations that would likely disrupt optimal substrate and cofactor interactions (Fig. 2D). Overall dynamic fluctuation profiles (RMSF per residue) further confirmed this pattern. The indel variant exhibited pronounced deviations from the wild-type profile in the region affected by the mutation, whereas all benign variants showed fluctuation patterns closely matching the wild-type (Fig. 2E). This pattern is also reflected in modestly lower Spearman and Pearson correlation coefficients between the wild-type and indel variant RMSF profiles relative to the benign controls (Table 1, columns 7 and 8). The reproducibility of the wild-type RMSF profile across the ten replicates is documented in Tables S2 and S3 (36) and provides an internal-consistency reference for these comparisons.

#### Inter-domain communication and substrate interactions

Because of the disrupted small-domain dynamics, we examined interaction energies between the protein and its ligands in the active closed model (Table 1, columns 9 and 10; Tables S2 and S3 (36)). Across the ten replicates, the indel variant showed a trend toward weaker ATP interaction relative to wild-type (Δ = +20.7 kcal/mol) that did not reach statistical significance (Welch t-test, *P* = .06), whereas its glucose interaction energy was essentially unchanged (Δ = +1.2 kcal/mol; *P* = .79). Among the pathogenic controls, only p.(Tyr108His) showed a significant reduction in ATP interaction (*P* = .004), and glucose interaction energies varied in both directions across benign and pathogenic variants (Tables S4 and S5 (36)). Perturbations in substrate interactions may therefore be deleterious in either direction, indicating that stronger interactions are not necessarily advantageous for these proteins; this is consistent with our previous studies of the KDM6A lysine demethylase and EHMT1 methyltransferase, in which proper substrate release following catalysis is essential for enzyme function (44, 45). Because interaction energies were calculated using an implicit-solvent model with unparameterized ligands, these values were considered exploratory rather than quantitative measures of binding. Similarly, distances between key catalytic elements showed bidirectional changes across all variants, including benign and pathogenic controls (columns 11-13), and therefore did not provide a consistent discriminatory pattern. This variability may reflect the absence of discrete active-site water molecules in the implicit-solvent model. In contrast, the clearest and most reproducible distinction was observed in the overall structural and dynamic metrics. The indel produced by far the largest global and local backbone perturbations (Table 1, columns 5 and 6) and the largest FoldX destabilization (column 4), and the FoldX ΔΔG values ordered the variants sensibly, with the benign controls near neutral, the pathogenic controls destabilizing, and the indel the most destabilizing of all. On this multi-layered analysis basis, the benign control variants consistently predicted all 3 benign control variants to be tolerated, whereas the pathogenic control variants and the indel variant were predicted to be damaging, in agreement with the GCK activity scores reported in the MAVE database (Table 1, column 3). These findings are also consistent with a recent comprehensive map of *GCK* variant effects generated by deep mutational scanning, which reported that hypoactive (functionally intolerant) variants concentrate at buried positions, near the active site, and at a region of known importance for GCK conformational dynamics within the small domain (46); the region affected by the indel variant described in this study lies within this conformational-dynamics region.

## Discussion

In this report, we describe a patient with suspected GCK-MODY who was previously diagnosed and treated as T2D. Genomic testing revealed a novel in-frame indel VUS in *GCK*, raising the suspicion of MODY2. Although her diabetes course was nonprogressive and otherwise consistent with MODY2, her clinical picture was complicated by a traumatic first pregnancy marked by pre-eclampsia and placental abruption, prompting aggressive glycemic control with insulin during her 2 subsequent pregnancies.

This case highlights 2 important and interrelated clinical challenges. First, GCK-MODY frequently goes undiagnosed before or during pregnancy, which can result in inadequate management. The hallmark features of GCK-MODY such as young age of onset, mild nonprogressive hyperglycemia, and absence of obesity can overlap significantly with gestational diabetes and early-onset T1D, leading to frequent misdiagnosis (21). In this case, a strong family history of early-onset diabetes, normal-weight BMI, biochemical findings that argued against autoimmune diabetes, and inadequate response to metformin and weight loss ultimately raised suspicion for monogenic etiology. However, because the identified GCK variant was classified as of unknown significance and given the limited understanding of its clinical implications at the time, her diabetes continued to be managed according to a T2D paradigm rather than one tailored to GCK-MODY.

Second, in pregnancies affected by GCK-MODY, the fetal genotype is typically unknown in utero, yet it has important implications for clinical management. Fetal pancreatic insulin secretion plays a central role in regulating fetal growth, and appropriate endogenous insulin levels are essential for normal development. When a mother with GCK-MODY carries an unaffected fetus, mild maternal hyperglycemia stimulates increased fetal insulin secretion, promoting insulin-mediated fetal overgrowth. Conversely, when the fetus inherits the *GCK* variant, the fetal glucose-sensing threshold is elevated, resulting in relatively normal insulin secretion and growth—particularly in the absence of pharmacological treatment of maternal hyperglycemia. Importantly, insulin treatment of the mother may paradoxically lead to fetal growth restriction when the fetus also carries the variant, as demonstrated in a study of 28 such pregnancies (47, 48).

In our case, given the patient’s anxiety following her complicated first pregnancy, insulin therapy was elected during the second and third pregnancies. Despite this, fetal growth remained within normal limits in both pregnancies, without evidence of growth restriction or other major complications. However, fetal genotyping was not performed in either pregnancy, which represents a limitation in fully interpreting growth trajectory considering the genotype-dependent risk described above.

Moreover, our structural modeling and MD simulations provide a mechanistic explanation for the deleterious effects of this variant over current sequence-based-only predictors. The introduction of a proline residue at position 105, combined with multiple amino acid substitutions and a single-residue deletion, leads to loss of β-strand integrity within the small domain and increased conformational variability during the transition to and within the active closed state. Unlike the wild-type and the benign control variants, which maintain stable and reproducible conformations across simulation replicates, the indel variant exhibits altered domain motions and reduced coordination of residue fluctuations. These perturbations are associated with protein destabilization, impaired inter-domain communication, and weakened interactions with ATP. These findings align with a recent comprehensive mapping of *GCK* variant effects by deep mutational scanning, which reported that hypoactive variants concentrate at buried positions, near the active site, and at a region important for GCK conformational dynamics of the small domain (46). The region affected by the indel variant in our study lies within this intolerant zone, reinforcing the functional relevance of the variant and highlighting the functional importance of the small domain regulatory region, beyond the catalytic center, in maintaining GCK activity. Several limitations of this study should be acknowledged. The MD simulations employed an implicit solvent model without additional parameterization of the ATP cofactor, Mg^2+^ ion, or glucose substrate. While this approach is appropriate for capturing relative differences in domain-scale dynamics and conformational sampling, it does not support quantitative interpretation of ligand-binding energetics or of the detailed solvent- and ion-mediated interactions within the active site. The cumulative simulation time of 100 ns per system, though augmented by ten independent replicates, may not fully capture slower conformational transitions. Additionally, no experimental enzymatic activity assay or thermal stability measurement was performed to validate computational predictions. Segregation analysis within the family was not available, and fetal genotyping was not performed, limiting the ability to correlate the variant with fetal growth outcomes.

## Conclusion

In conclusion, we describe a novel in-frame indel variant in GCK identified in a woman with clinical features consistent with GCK-MODY and managed across 2 pregnancies. Structural modeling and MD simulations indicate that the variant destabilizes the small domain and disrupts its coordinated motion, with associated impairment of inter-domain communication and a trend toward weaker ATP interaction. Together these findings provide mechanistic support for a loss-of-function effect and highlight the functional importance of the small domain conformational-dynamics region, beyond the catalytic center in maintaining GCK activity. This work expands the known genotypic spectrum of GCK-MODY and provides clinical management and mechanistic framework that may contribute to the reclassification of the novel indel variant.

## Data Availability

The molecular dynamics datasets generated and analyzed in this study are publicly available in MDRepo under accession numbers MDR00058442, MDR00058456, MDR00065536, and MDR00065725 (https://mdrepo.org/explore?created_by_id=250). Additional datasets, including starting structures and analysis settings, are available from the corresponding author on reasonable request.
